# Fusion Imaging of X-ray and Transesophageal Echocardiography Improves the Procedure of Left Atrial Appendage Closure

**DOI:** 10.1007/s10557-020-07048-z

**Published:** 2020-08-06

**Authors:** Henning Ebelt, Thomas Domagala, Alexandra Offhaus, Matthias Wiora, Andreas Schwenzky, Matthias Hoyme, Jelena Anacker, Peter Röhl

**Affiliations:** Department for Medicine II, Catholic Hospital “St. Johann Nepomuk”, Haarbergstr. 72, 99097 Erfurt, Germany

**Keywords:** Atrial fibrillation, LAAC, Fusion imaging

## Abstract

**Background:**

Left atrial appendage closure (LAAC) is an alternative treatment strategy for patients with atrial fibrillation who are at risk for thromboembolic events and considered not suitable for oral anticoagulation (OAC). LAAC is mainly performed under the guidance of transesophageal echocardiography (TEE) and fluoroscopy. The study presented here should analyze whether fusion imaging (FI) of transesophageal echocardiography and X-ray performed during LAAC is feasible and can improve the results of the procedure.

**Methods:**

The data presented here are from a retrospective single center study. Sample size was defined as 50 patients in which LAAC was performed without fusion imaging (control group) and 25 patients were the LAAC procedure was guided by fusion imaging (treatment group). Inclusion criteria were defined as age > 18 years and completion of an LAAC procedure defined as deployment of a WATCHMAN 2.5 LAA occluder. Study endpoints were procedure time, amount of used contrast medium, radiation dose, final position of the WATCHMAN in TEE (deviation from ideal positioning), and clinical endpoints, respectively.

**Results:**

LAA closure was successfully performed in all patients. No case of device embolism was occurring, and none of the patients experienced a periprocedural stroke/TIA nor a systemic embolism, respectively. Mean procedure time was 15 min shorter in the group of patients where fusion imaging was applied (*p* < 0.001). Additionally, the use of fusion imaging was associated with a significant reduction of contrast medium (20.6 ml less than in control; *p* < 0.045). Regarding the final position of the WATCHMAN, no relevant differences were found between the groups.

**Summary:**

The use of fusion imaging significantly reduced procedure time and the amount of contrast medium in patients undergoing LAAC.

## Background

Atrial fibrillation (AF) carries the risk of intracardiac thrombus formation and embolic stroke. Oral anticoagulation (OAC) is the standard of care in patients with AF who are at increased thromboembolic risk [1]. However, there are a certain number of patients who have either experienced or are considered to be at increased risk of bleeding complications under OAC, respectively, and who therefore are candidates for occlusion of the left atrial appendage (LAA) as an alternative treatment strategy. In the vast majority of cases, LAA closure (LAAC) is performed by a transfemoral approach under the simultaneous guidance of both transesophageal echocardiography (TEE) and fluoroscopy, whereas alternative imaging modalities like intracardiac ultrasound (ICE) are used only in a minority of cases but with similar procedural results [2].

In daily practice, ultrasound and X-ray are normally displayed independently on different screens so that the operator has to “fuse” the information of both modalities mentally. Nevertheless, in contemporary cath labs, the technology of dynamic fusion imaging of TEE and fluoroscopy is in principle available but is rarely used which is in part due to limited experience and the lack of data showing a clinical benefit of this technology. The study presented here should analyze whether fusion imaging (FI) of transesophageal echocardiography and X-ray performed during LAAC is feasible and can improve the results of the procedure.

## Methods

The data presented here are derived from a retrospective single center study performed at the Catholic Hospital “St. Johann Nepomuk”, Erfurt, Germany. The ethics committee of the medical association of Thuringia approved this analysis.

Patients in which a left atrial appendage closure was performed before December 01, 2019, were retrospectively screened for inclusion into this study (retrospective consecutive approach). Sample size was defined as 50 patients in which LAAC was performed without fusion imaging (control group) and 25 patients were the LAAC procedure was guided by fusion imaging (treatment group). Inclusion criteria were defined as age > 18 years and completion of an LAAC procedure defined as deployment of an LAA occluder. The only exclusion criterium was the use of another LAA occluder than WATCHMAN 2.5 in order to allow a standardized echocardiographic quantification and comparison of the implantation result. All LAAC procedures were done by the same team of experienced operators that all have been performing LAAC since 2014. In the current study, the procedures without FI were performed between November 16, 2016, and January 29, 2019, and the procedures with FI between October 28, 2018, and November 05, 2019.

### LAA Closure Without Fusion Imaging (Control Group)

According to local standards, patients were kept under conscious sedation induced by 5 mg i.v. midazolame and 0.1 mg i.v. fentanyl, additional fentanyl or etomidate could be added if considered necessary. After local anesthesia, venous access was established using the right femoral vein and transseptal puncture (TSP) was performed under separate guidance of both fluoroscopy (Artis zee, Siemens Healthineers, Erlangen, Deutschland) and TEE (either ACUSON SC2000, Siemens Healthineers, Erlangen, Deutschland, or Vivid E9, General Electrics, Boston, MA, USA, respectively). Anticoagulation with a target activated clotting time (ACT) of 250–300 s was achieved by i.v. administration of a bolus of unfractionated heparin (7500 to 10,000 IU), ACT was determined in 20 min intervals until the end of the procedure. After TSP, an Amplatzer Super Stiff wire was placed into a left pulmonary vein and the TruSeal WATCHMAN sheath was brought into the left atrium (LA). A 5F pigtail catheter was placed into the LAA and one angiographic projection of the LAA was recorded (standard: RAO 30/ caudal 30); if considered necessary by the implanting physician, an additional angiographic view could be taken. The dimension of the LAA ostium and the occluder landing zone was determined both by TEE (0°, 45°, 90°, and 135°) and by angiography and the size of the LAA occluder was selected according to the instructions for use. Before the LAA occluder was permanently delivered, all standard criteria (position, anchoring, sizing, seal) were checked by TEE. The final implantation result was documented by TEE and angiography.

### LAA Closure Under Guiding of Fusion Imaging (Treatment Group)

**Fig. 1:**
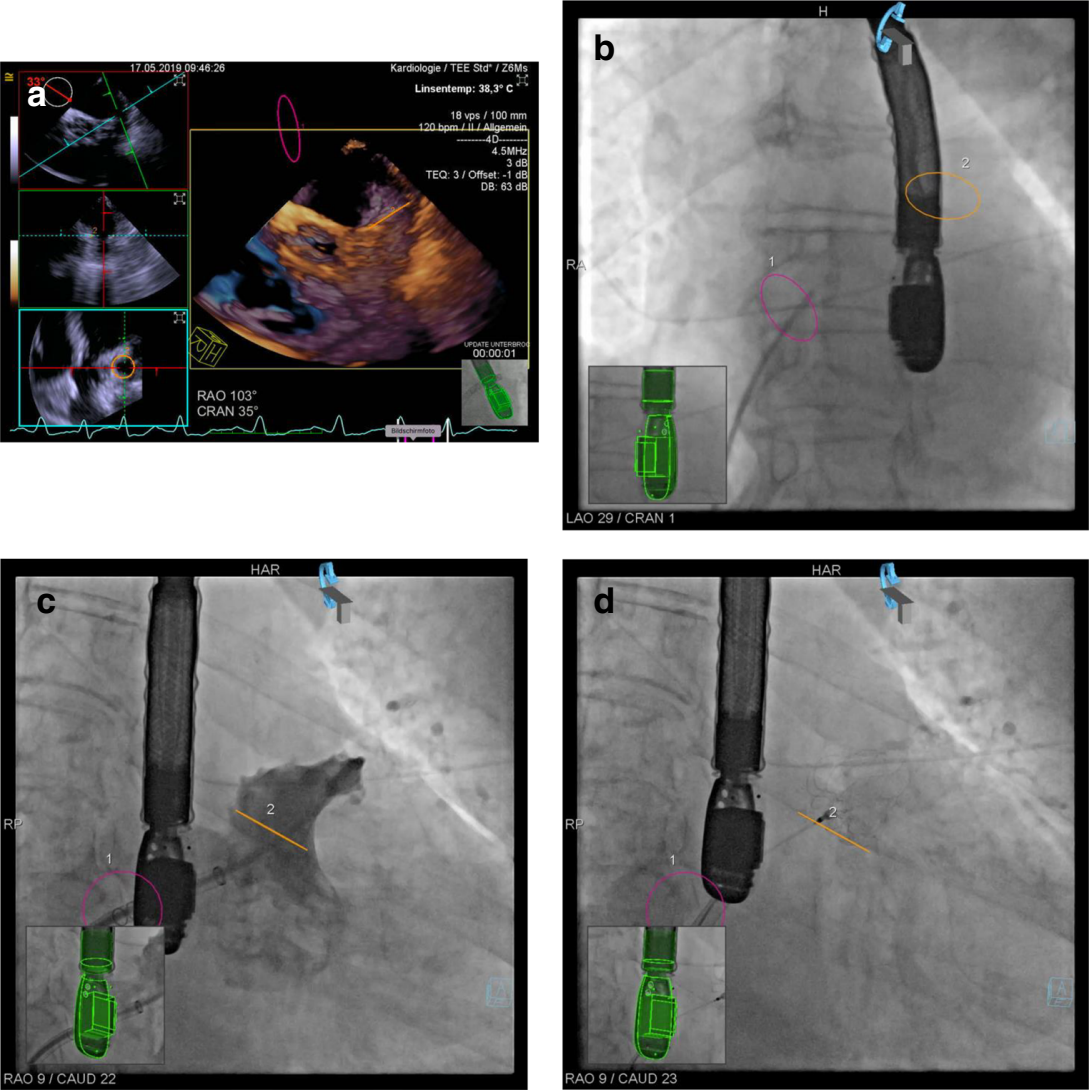
Example of left atrial appendage closure guided by fusion imaging. (A) Placement of circle 1 (fossa ovalis, purple color) and circle 2 (LAA ostium, orange color) in TEE. (B) Preparation of transseptal puncture, transseptal needle in fossa ovalis (circle 1, LAO projection). (C) Angiography of LAA in patient-specific C-arm angulation at which the LAA ostium is shown in an orthogonal projection (circle 2 is displayed as a line). (D) Final position of the LAA occluder (WATCHMAN 2.5)

Fusion imaging (FI) was performed using TrueFusion technology (Siemens Healthineers, Erlangen, Deutschland). According to local standards, FI-guided LAAC was modified at several steps in comparison with the control group. First, co-registration of angiography (Artis zee, Siemens Healthineers, Erlangen, Deutschland) and TEE (ACUSON SC2000, Siemens Healthineers, Erlangen, Deutschland) was started using the dedicated “eSie Sync” algorithm. After venous access, both the fossa ovalis (circle 1) and the LAA ostium (circle 2) were marked by TEE and then sent to the fluoroscopic imaging system (Fig. 1A). TSP was performed as in control group but with the additional information of the oval fossa (circle 1) displayed on the fluoroscopic screen (Fig. 1B). After TSP, FI was used to find a patient-specific fluoroscopic projection at which the LAA ostium was visualized orthogonal (which means that the corresponding circle 2 was displayed as a line). Both the angiography of the LAA and all following steps were performed at this individualized C-arm angulation (Fig. 1 C, D). A summary of the specific protocol used for FI-guided LAAC is given in Table 1.

**Table 1:** Protocol for fusion imaging–guided closure of the left atrial appendage

Start of fusion imaging (FI, TrueFusion) by co-registration of angiography and TEE (automatic probe detection, “eSie Sync” algorithm)	
TEE: delineation of fossa ovalis (circle 1) and LAA ostium (circle 2); transfer of these labels to fluoroscopic imaging system (Artis zee)	
Transseptal puncture according to standard techniques using FI as supporting modality (circle 1)	
FI-guidance during occluder implantation:	
Placement of 5F pigtail catheter into the LAA (circle 2)	
Identification of optimal patient-specific C-arm angulation (orthogonal projection of LAA ostium, circle 2)	
Verification of correct occluder positioning during release procedure (circle 2)	
Completion of LAAC according to standard procedures using FI as supporting modality	

### Data Capture

**Fig. 2:**
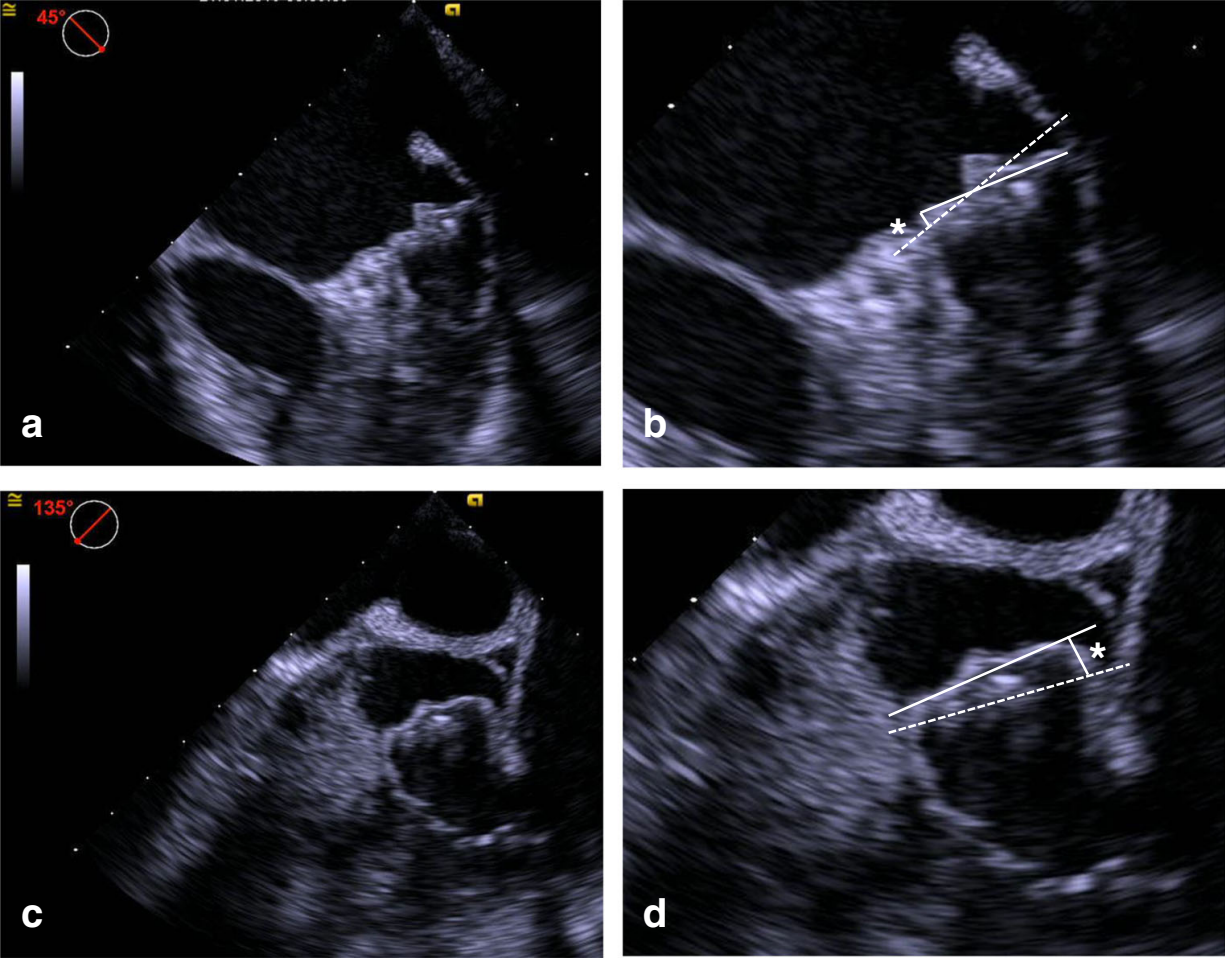
Example of the evaluation of final position of the LAA occluder (WATCHMAN 2.5) in TEE. Images (B) and (D) are showing sections of images (A) and (C), respectively. The “ideal” position of the WATCHMAN occluder (landing zone) is marked by the dotted line, which was used as a reference for the TEE measurements; the asterisk is indicating the maximum of deviation of the occluder towards the LA

All patient-related data were retrospectively extracted from the institutional database. Data describing details of the implantation procedure (amount of contrast medium, radiation dosage) were taken from cath lab reports, and procedure time was defined as the time between femoral venous puncture and final removal of the femoral sheath. All study-specific measurements from angiographic or TEE images were performed by a blinded operator. Therefore, all images were pseudonymized and all recordings which were containing information indicative for the use of FI were removed in order to minimize potential bias during the following quantifications. From the angiographic recordings, the number of complete device recaptures was counted and the size (compression) of the LAA occluder at its final position was measured. From the TEE sequences, the following measurements were taken after the final device deployment at 0°, 45°, 90°, and 135°: device protrusion towards the left atrium, size of paradevice leakage, device size (compression), angle between device front surface and LAA ostial plane (Fig. 2).

### Statistics

Statistical data analysis was performed using SPSS Statistics (version 26.0, SPSS Inc., IBM, Armonk, NY). Metric variables are expressed as mean ± standard deviation, and Student’s *t* test was used to analyze differences between control and treatment group. Differences in the frequency of nominally scaled parameters were compared by means of Pearson‘s chi-squared test or Fisher’s exact test. The basis for the test decisions was a significance level of *p* < 0.05.

## Results

Baseline parameters of the included patients are given in Table 2; no relevant disparities were found between the groups.

**Table 2:** Demographic parameters of patients with atrial fibrillation at baseline (before LAA closure). *AF*. atrial fibrillation; *CAD*, coronary artery disease; *GFR*, glomerular filtration rate

Parameter	Control (*N* = 50)	Fusion imaging (*N* = 25)	*p* value
Male sex	25 (50%)	11 (44%)	0.81
Age [years]	75.3 ± 8.9	77.2 ± 6.3	0.35
Height [cm]	167.8 ± 8.7	168.3 ± 9.8	0.80
Weight [kg]	82.1 ± 19.4	80.7 ± 17.7	0.78
GFR [ml/min*1.73m^2^]	65.5 ± 31.1	53.2 ± 19.9	0.08
CHA2DS-VASc	4.3 ± 1.2	4.5 ± 1.5	0.53
HASBLED	2.9 ± 0.9	2.9 ± 0.9	1.00
Type of AF			0.67
Paroxysmal	24 (48%)	10 (40%)	
Persistent	8 (16%)	6 (24%)	
Permanent	18 (36%)	9 (36%)	
Hypertension	47 (94%)	20 (80%)	0.11
Diabetes	20 (40%)	11 (44%)	0.81
History of stroke	12 (24%)	6 (24%)	1.00
CAD	21 (42%)	14 (56%)	0.33

According to the study inclusion criteria, LAA closure was successfully performed in all patients. During hospital stay, no case of device embolism was occurring, and none of the patients experienced a periprocedural stroke/TIA nor a systemic embolism, respectively. There were 3 cases of pericardial effusion which were successfully treated by percutaneous drainage (Table 3).

**Table 3:** Clinical outcomes of patients with atrial fibrillation and LAA closure depending on the use of fusion imaging during the intervention. *TIA*, transitoric ischemic attack; *LAA*, left atrial appendage

Parameter	Control (*N* = 50)	Fusion imaging (*N* = 25)	*p* value
Death	0 (0%)	0 (0%)	NA
Stroke/TIA	0 (0%)	0 (0%)	NA
Systemic embolism	0 (0%)	0 (0%)	NA
Pericardial effusion	3 (6%)	0 (0%)	0.55
Device embolization	0 (0%)	0 (0%)	NA

As seen in Table 4, several differences between the groups could be found regarding the technical details of the implantation procedure. Mean procedure time was 15 min shorter in the group of patients where fusion imaging was applied (*p* < 0.001). In line with this finding, the number of complete recapturing maneuvers of the LAA occluder was also significantly reduced in the FI group. Additionally, the use of fusion imaging was associated with a significant reduction of contrast medium (20.6 ml less than in control; *p* < 0.045).

**Table 4:** Procedural parameters of interventional left atrial appendage closure depending on the use of fusion imaging

Parameter	Control (*N* = 50)	Fusion imaging (*N* = 25)	*p* value
Procedure time [min]	45.2 ± 19.9	30.2 ± 9.1	< 0.001
Contrast volume [ml]	69.3 ± 42.0	48.7 ± 30.6	0.03
Radiation dose [Gycm^2^]	27.6 ± 57.8	14.0 ± 7.9	0.25
Number of devices used	1.1 ± 0.4	1.1 ± 0.4	0.87
Number of complete device recaptures	1.1 ± 1.5	0.4 ± 0.9	0.01
Size of implant [mm]	26.9 ± 3.5	26.6 ± 3.6	0.73
Device compression in fluoroscopy [%]	11.6 ± 8.2	10.6 ± 7.8	0.65

Regarding the final position of the WATCHMAN occluder, no relevant differences were found between the groups. Both the size of the implanted occluders and the compression rate were comparable in both groups (Table 4), and detailed quantification of the TEE images did not reveal any distinctive feature (Table 5).

**Table 5:** TEE evaluation of final result of left atrial appendage closure depending on the use of fusion imaging

Parameter (TEE)	Control (*N* = 50)	Fusion imaging (*N* = 25)	*p* value
Max. protrusion towards LA [mm]	9.6 ± 4.8	10.3 ± 2.9	0.44
Max. deviation of device from LAA ostium plane [degree]	13.1 ± 11.5	16.0 ± 4.9	0.13
Device compression [%]			
TEE 0°	24.3 ± 11.1	20.7 ± 9.8	0.24
TEE 45°	24.5 ± 9.1	23.1 ± 9.1	0.55
TEE 90°	25.0 ± 8.1	19.8 ± 13.1	0.08
TEE 135°	24.2 ± 8.0	22.4 ± 11.8	0.54
Any paradevice leak	2 (4%)	1 (4%)	1.00
Leak < 3 mm	2 (4%)	1 (4%)	1.00
Leak ≥ 3 mm	0 (0%)	0 (0%)	NA

## Discussion

Availability and the adequate use of different imaging modalities are key prerequisites for the majority of structural interventions in cardiology. Due to the ongoing improvements in catheter-based technologies and the rising need for less invasive cardiovascular interventions, the number and complexity of structural procedures has been rising for years. In a relevant number of cases, these interventions are performed under the simultaneous guidance of more than one imaging modality. From a theoretical perspective, it should be an advantage to combine and fuse the complementary information of different imaging modalities like ultrasound and fluoroscopy instead of only simultaneously displaying these images on separate screens. Therefore, several technical solutions for fusion imaging have been established that are nowadays commercially available [3–5]. However, to date, there are only a very limited number of studies which have shown the feasibility and safety of dynamic fusing imaging of TEE and fluoroscopy during cardiac interventions [6], and only one small study with a number limitations has pointed out towards a clinical advantage of the use of FI during interventional LAA closure [7].

At our study, a standardized protocol has been applied to include FI into the LAAC workflow. During TSP, FI can supply additional information of the localization and spatial orientation of the oval fossa if displayed on the fluoroscopic screen. It has been reported that this can lead to a faster TSP [8] and can be considered especially helpful in challenging cases (e.g., patients after cardiac surgery; presence of implanted devices) [9].

Our analysis shows that the integration of FI into a standardized workflow leads to reduced procedure time and a reduction of X-ray contrast medium in LAAC which is of clinical relevance both from the patients and from the interventionalist’s perspective. It is important to note that in the control group of our study, the procedural data are very much in line with previous reports thereby arguing for a real “improvement” of the procedure in the treatment group (FI) rather than a “poor” control group. As published by Reddy et al. in 2017 [10], in 3822 procedures in the post-approval situation of the WATCHMAN occluder in the USA, the mean procedure time was 50 min (IQR: 36–56 min) which is very close to the time of control patients in our study (45.2 ± 19.9 min), and we could show that this time was reduced by 33% by the use of FI. Likewise, in recent trials, the amount of X-ray contrast agent used during LAAC was reported to be 90.0 ± 64 ml [11] or 70 ± 20 ml [12], respectively. Again, these data are very much reflecting the situation found in our control group (69.3 ± 42 ml) which in parallel argues for a “real” reduction of contrast medium use in our study if fusion imaging was used (48.7 ± 30.6 ml). An explanation for these improvements can be seen in the fact that the use of FI leads to an individualized, patient-specific fluoroscopic imaging so that a number of additional X-ray projections which are otherwise often necessary to find the “optimal” C-arm position are dispensable.

One previous paper also reported positive effects of FI in LAAC in regard to radiation dose and fluoroscopy time [7]. However, both procedure time (90.1 ± 30.2 min) and the amount of contrast agent (197.5 ± 127.8 ml) were surprisingly high in control patients in this study and therefore considered a source of concern [7].

It is noteworthy that in our study, the number of patients with pericardial effusion (PE) was numerically lower in the group that underwent LAAC with FI. Although this difference did not reach statistical significance, there are several arguments suggesting that the use of FI could increase the safety of LAAC. As stated above, transseptal puncture (TSP) which always contains some risk for PE is supported by FI so that FI might reduce the risk of TSP-related PE. Additionally, recapturing maneuvers of the LAA occluder which are necessary when the position of the device is considered sub-optimal are also known to contain a risk of PE and our study has shown that the number of complete device recaptures was reduced by FI by more than 60% (see Table 4).

### Limitations

Several constraints of our study have to be considered. First, the number of patients is limited and all data are derived from a retrospective analysis and not from a prospective randomized trial so that it cannot be ruled out that some kind of selection bias has occurred. However, a randomized study on that topic could not be performed in a blinded fashion thereby carrying the risk of operator bias. Additionally, although all LAAC have been performed by experienced operators, the time period of cases done with FI started later than that of the conventional procedures. Furthermore, we only show data regarding the use of one dedicated LAA occluder (WATCHMAN 2.5) and one fusion imaging system (Siemens TruFusion) so it is not clear whether the results are also valid for other LAAC devices and FI systems, respectively. Follow-up of the patients in our study was limited to the hospital stay of the index procedure with no further imaging at later time points.
