# Correction to: Fusion Imaging of X-ray and Transesophageal Echocardiography Improves the Procedure of Left Atrial Appendage Closure

**DOI:** 10.1007/s10557-020-07102-w

**Published:** 2020-10-27

**Authors:** Henning Ebelt, Thomas Domagala, Alexandra Offhaus, Matthias Wiora, Andreas Schwenzky, Matthias Hoyme, Jelena Anacker, Peter Röhl

**Affiliations:** Department for Medicine II, Catholic Hospital “St. Johann Nepomuk”, Haarbergstr. 72, 99097 Erfurt, Germany

**Correction to: Cardiovascular Drugs and Therapy (2020)**

10.1007/s10557-020-07048-z

The article “Fusion Imaging of X-ray and Transesophageal Echocardiography Improves the Procedure of Left Atrial Appendage Closure” Henning Ebelt, Thomas Domagala, Alexandra Offhaus, Matthias Wiora, Andreas Schwenzky, Matthias Hoyme, Jelena Anacker, and Peter Röhl was originally published Online First without Open Access. After publication, the author decided to opt for Open Choice and to make the article an Open Access publication. Therefore, the copyright of the article has been changed to © The Author(s) 2020 and the article is forthwith distributed under the terms of the Creative Commons Attribution 4.0 International License (http://creativecommons.org/licenses/by/4.0/), which permits use, duplication, adaptation, distribution, and reproduction in any medium or format, as long as you give appropriate credit to the original author(s) and the source, provide a link to the Creative Commons license, and indicate if changes were made.

The original article has been corrected.

Open Access This article is distributed under the terms of the Creative Commons Attribution 4.0 International License (http://creativecommons.org/licenses/by/4.0/), which permits unrestricted use, distribution, and reproduction in any medium, provided you give appropriate credit to the original author(s) and the source, provide a link to the Creative Commons license, and indicate if changes were made. The images or other third party material in this article are included in the article’s Creative Commons license, unless indicated otherwise in a credit line to the material. If material is not included in the article’s Creative Commons license and your intended use is not permitted by statutory regulation or exceeds the permitted use, you will need to obtain permission directly from the copyright holder. To view a copy of this license, visit http://creativecommons.org/licenses/by/4.0/.

